# Supplementary material for: Ocean warming drives immediate mass loss from calving glaciers in the high Arctic
Source: Nat Commun. 2024 Dec 2;15:10460. doi: 10.1038/s41467-024-54825-7 (PMC11612468; doi:10.1038/s41467-024-54825-7)
Supplement: Supplementary file 1 — Supplementary Information [file 41467_2024_54825_MOESM1_ESM.pdf]

Supplementary materials for the article *"Ocean warming drives immediate mass loss from calving glaciers in the high Arctic"* by Foss et al.

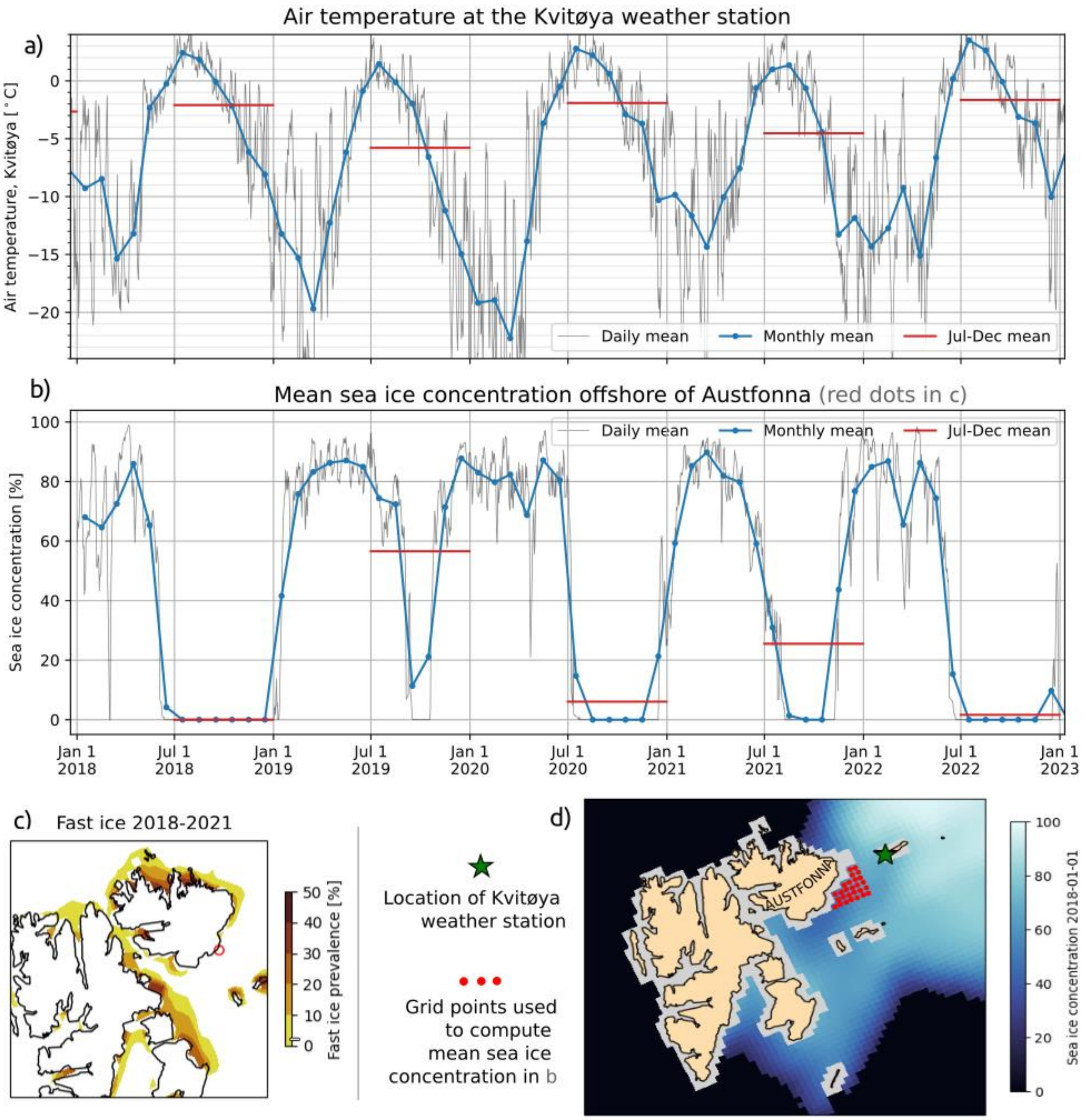

Supplementary Figure S1. Environmental conditions during the study period. a) Air temperature at the Kvitøya weather station [1]. b) Average sea ice concentration offshore of eastern Austfonna, in the northwesternmost Barents Sea. Sea ice concentration data obtained from [2]. c) Percentage of data points 2018-2021 (at ~weekly resolution) classified as "fast ice" in [3]. d) Location of the Kvitøya weather station and the grid points used to compute the mean sea ice concentration. Underlying colors show sea ice concentration from [2] on 01.01.2018.

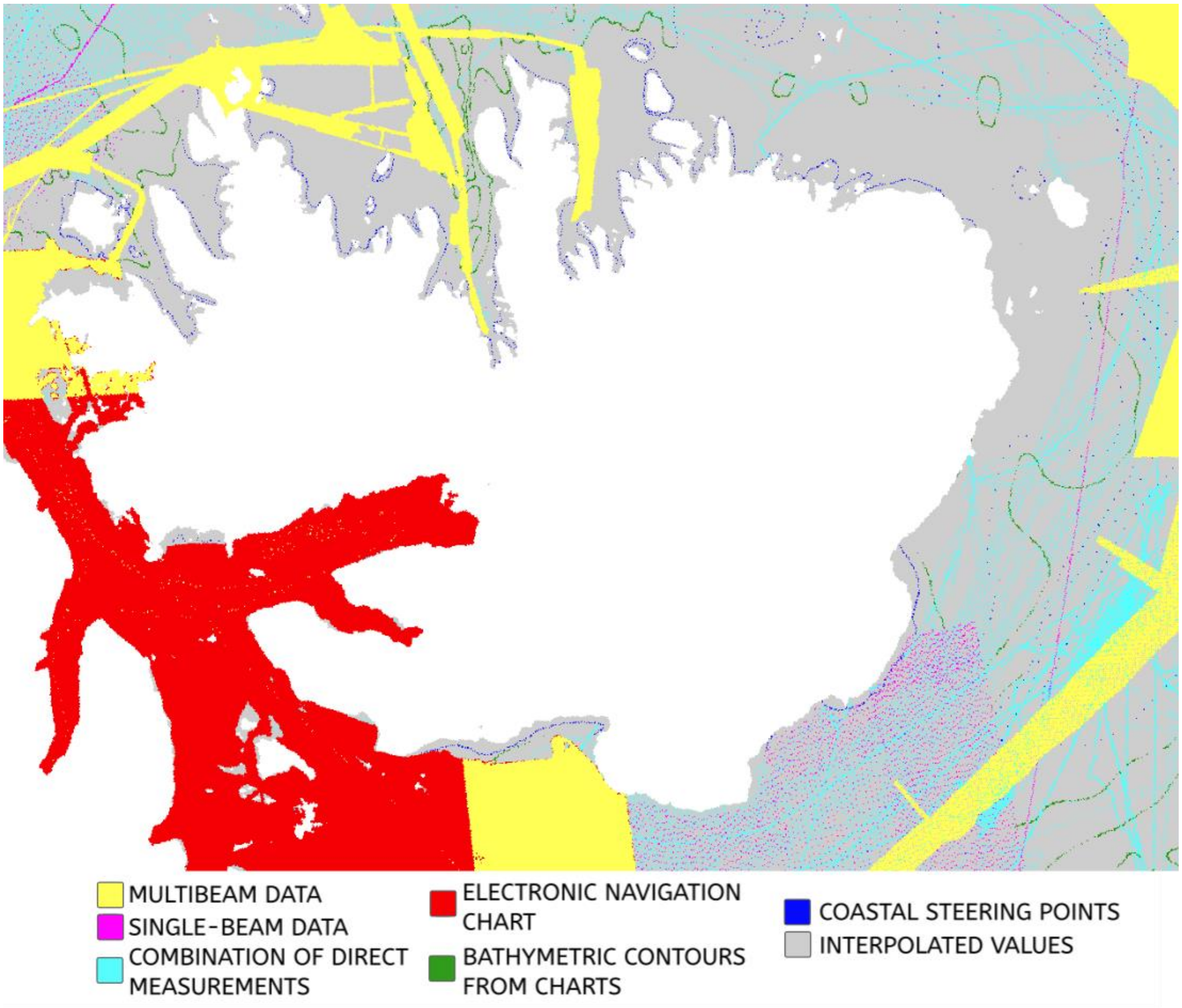

Supplementary Figure S2. Map of source data for IBCAO v4[4] bathymetry around Nordaustlandet. From the IBCAO v4 200m x 200m Data Type Identifier Grid. This map was used to generate the shaded contours in Fig. 1e, indicating areas with no underlying source data (interpolated values) or areas where only coastal "steering points" have been used. The latter assumes zero depth at the coastline, which may not be a good approximation at glacier margins. These data were not used in the glacier-ocean bedrock compilation used in the frontal ablation calculations (see Methods).

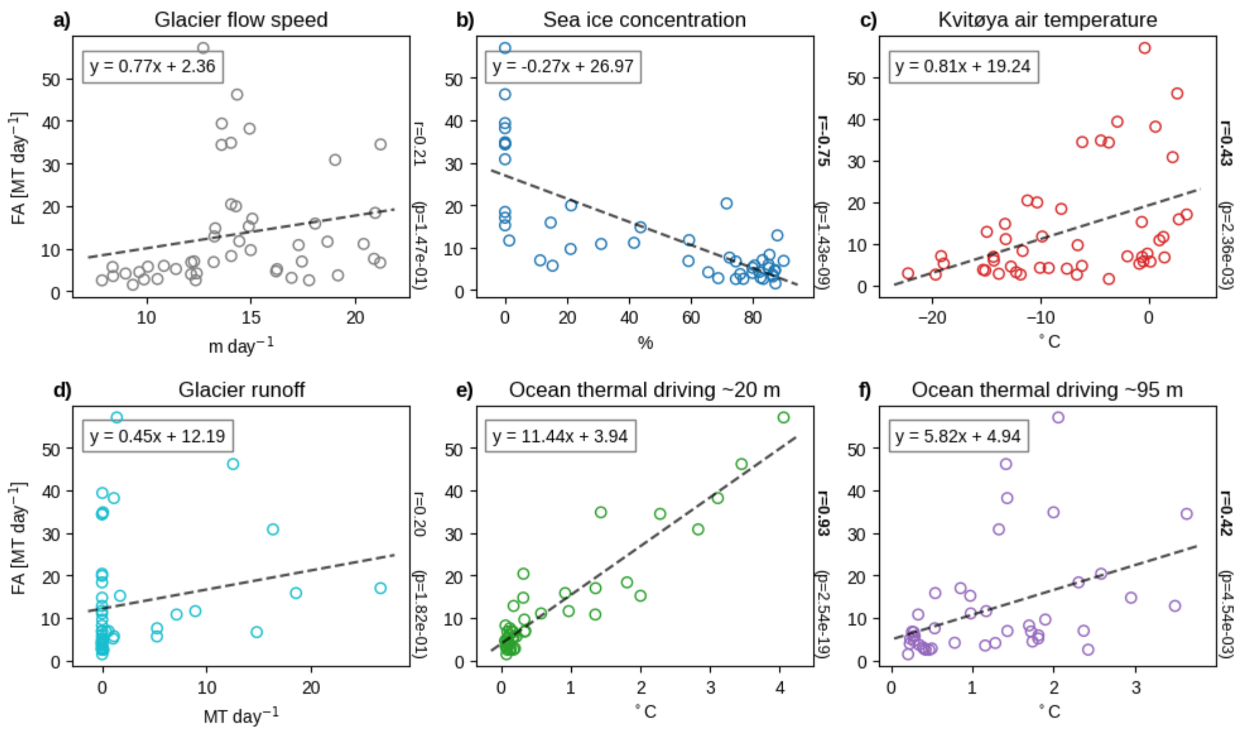

Supplementary Figure S3. Scatter plots of monthly averaged frontal ablation at Storisstraumen versus monthly averaged environmental parameters: a) Glacier flow speed (Fig. 2a), b) Offshore sea ice concentration (Fig. S1b, d), c) Air temperature at Kvitøya (Fig. S1a, d), Modelled glacier runoff (Fig. 2b). Panels e and f show the thermal driving (ocean temperature in degrees above freezing) from the ocean mooring near ~95 m and ~20 m, respectively (Fig. 1c). All quantities have been resampled to monthly averages (see Methods). Dashed lines show linear fits, with the linear parameters shown in the inset text box. Pearson‘s correlation coefficients and associated p-values are shown on the right edge of each panel. Significant ($p<0.05$) correlations are written in bold type. Correlations and fits exclude data gaps.

References

1. MET Norway. Observations from station KVITØYA (SN99938). Obtained from MET Norway data portal www.seklima.met.no.
2. EUMETSAT Ocean and Sea Ice Satellite Application Facility. OSI SAF Global Sea Ice Concentration (SSMIS), OSI-401-d [Data Set] [Data extracted from OSI SAF Thredds]. https://doi.org/10.15770/EUM_SAF_OSI_NRT_2004.
3. U.S. National Ice Center (Fetterer, F. & Stewart, J. S. (Comps.)). U.S. National Ice Center Arctic and Antarctic Sea Ice Concentration and Climatologies in Gridded Format, Version 1. National Snow and Ice Data Center https://doi.org/10.7265/46cc-3952 (2020).
4. Jakobsson, M. *et al.* The International Bathymetric Chart of the Arctic Ocean Version 4.0. *Sci. Data* **7**, 176 (2020).
